# Effects of Unsuppressed Endogenous Insulin on Pharmacokinetics and/or Pharmacodynamics of Study Insulin in the Healthy: A Retrospective Study

**DOI:** 10.1002/cpdd.1093

**Published:** 2022-04-05

**Authors:** Hui Liu, Hongling Yu, Lisi Sun, Jingtao Qiao, Jiaqi Li, Huiwen Tan, Yerong Yu

**Affiliations:** ^1^ Department of General Practice West China Hospital Sichuan University Chengdu Sichuan China; ^2^ Department of Endocrinology and Metabolism West China Hospital Sichuan University Chengdu Sichuan China

**Keywords:** C‐peptide, endogenous insulin secretion, euglycemic clamp, insulin pharmacokinetics, insulin pharmacodynamics

## Abstract

C‐peptide, a marker of endogenous insulin, should be consistently inhibited during euglycemic clamping, while an elevated postdosing C‐peptide (CP_postdosing_) is not an occasional phenomenon. This was a retrospective study that included 33 men who underwent a manual euglycemic clamp with a subcutaneous injection of insulin aspart (IAsp) aiming to describe the effects of insufficient suppression of endogenous insulin on estimates of the pharmacokinetics and pharmacodynamics of injected insulin. The time profiles of whole blood glucose, human insulin, glucose infusion rate (GIR), and C‐peptide were recorded. The subjects were divided into 2 groups at a ratio of 2:1: group A ([CP_postdosing_]_max_>baseline CP [CP_baseline_]), group B ([CP_postdosing_]_max_ ≤ CP_baseline_). The endogenous insulin was approximately equal to the measured value of human insulin or calculated from the C‐peptide. The basal glucose, CP_baseline_, basal human insulin, homeostatic model assessment of insulin resistance, IAsp dose, and demographic statistics were all comparable between the 2 groups except the “clamped” glucose. The average clamped glucose was 99.7% (group A) and 94.9% (group B) of baseline. After correction for clamped glucose, GIR area under the concentration‐time curve from time 0 to 8 hours was higher in group A (*P* < .05) under comparable IAsp exposure. Endogenous insulin area under the concentration‐time curve from time 0 to 8 hours calculated from C‐peptide was different from that measured from human insulin in group A (*P* < .05), whereas no statistical difference between these measures was observed in group B. Hence, blood glucose should be controlled below the baseline to ensure the inhibition of endogenous insulin. Unsuppressed endogenous insulin may contribute to observed GIR, and the endogenous insulin–corrected pharmacokinetics estimated by C‐peptide may be inaccurate with insufficient endogenous insulin suppression.

In euglycemic clamp studies aimed at evaluating the pharmacokinetics (PK) and pharmacodynamics (PD) of new insulin preparations in healthy subjects, “clamped” blood glucose levels have been found to be below the subjects’ basal glucose (eg, 10% or 5 mg/dL lower than fasting glucose levels)^1^, ^2^, ^3^, ^4^, ^5^ or to remain around baseline.^6^, ^7^, ^8^, ^9^, ^10^, ^11^, ^12^, ^13^ A priming dose of rapid‐acting insulin followed by a basal rate of intravenous insulin infusion (eg, 0.1‐0.15 mU/min/kg)^14^, ^15^, ^16^, ^17^ can be used, but the latter has been largely abandoned since the finding that the effect of such infusion increases over time.^18^ The sufficient inhibition of endogenous insulin in such studies is of paramount importance, and C‐peptide should always be measured in parallel to exogenous insulin concentration to estimate the extent and consistency of the suppression of endogenous insulin throughout the experiment. During euglycemic clamp procedures that use an insulin analog, if PK‐specific assays for the analog are lacking, the ratio of insulin to C‐peptide before dosing can be used to distinguish it from native human insulin in serum assays.^4^, ^6^, ^7^, ^19^ However, we noticed that C‐peptide levels can be higher than the baseline values after the administration of exogenous insulin during the clamp procedure. Other researchers have reported similar observations.^8^, ^9^

Here, we report the time profiles of insulin aspart (IAsp), human insulin, and C‐peptide after a subcutaneous injection of IAsp during an 8‐hour manual euglycemic clamp procedure conducted in healthy Chinese male volunteers. The main aim was to determine the effects of elevated postdosing C‐peptide on estimates of PD and/or PK (when using C‐peptide to correct endogenous insulin in the absence of PK‐specific assay) of the insulin analog. The second aim was to explore the consistency of the changes in C‐peptide and endogenous insulin levels during the euglycemic clamp.

## Methods

This study was a retrospective study, and we collected the data from the database of 2 clinical trials (CTR20160095, CTR20180517) performed from 2016 to 2018. These 2 clinical trials aimed to evaluate the PK and PD similarity of the IAsp biosimilar produced by Zhuhai United Laboratories Co., Ltd,^20^ and Yichang HEC Changjiang Pharmaceutical Co., Ltd,^21^ respectively, with NovoRapid. In the present study, the subjects would be assigned to group A if the highest postdosing C‐peptide ([CP_postdosing_]_max_) level was higher than basal C‐peptide (CP_baseline_), and the subjects whose (CP_postdosing_)_max_ level did not exceed baseline would be allocated to group B. The ratio of the sample size of group A to that of group B was 2:1.

### Participants

Healthy Chinese men between 18 and 45 years of age with a body mass index between 18 and 24 kg/m^2^ were considered eligible. All participants were nonsmokers and without a family history of diabetes mellitus or hypertension. Their blood glucose concentrations were within a normal range (fasting glucose level <110 mg/dL, 2‐hour 75‐g oral glucose tolerance test <140 mg/dL, and glycosylated hemoglobin <6.1%), and no abnormalities were found in an electrocardiogram, a complete blood count and urinalysis, or liver and renal function.

The trial procedures were carried out in accordance with the Declaration of Helsinki and the principles of Good Clinical Practice. All participants provided informed consent, and the study was approved by the ethics committee of West China Hospital of Sichuan University.

### Euglycemic Clamp Procedure

From 3 days before the dosing day on, participants were instructed to abstain from drinking alcohol, smoking tobacco, engaging in strenuous exercise, and ingesting caffeine. Participants came to the research center at approximately 6:00 pm the day before drug administration to ensure an overnight fasting condition of 10 to 12 hours the next morning. Throughout the clamp, the subjects remained fasting and in a supine position. A 20‐gauge polyethylene cannula was inserted into an antecubital vein for infusion of 20% dextrose, and a second 18‐gauge catheter was inserted retrogradely into a wrist vein on the dorsum of the hand to draw blood. The hand for blood drawing was maintained continuously in a heated blanket at 55 to 65°C, allowing sampling of arterialized venous blood. After recording the basal blood glucose level (defined as the mean of the glucose measurement at −30, −20, and −10 minutes), the subjects received a 0.2 IU/kg dose of NovoRapid (Novo Nordisk, Bagsværd, Denmark) by subcutaneous injection into a lifted abdominal skin fold. Blood samples were obtained at the bedside for immediate determination of whole blood glucose concentrations every 5 minutes from 0 to 240 minutes and every 10 minutes from 240 to 480 minutes. During the manual euglycemic clamp procedure, the glucose infusion rate (GIR) was adjusted on the basis of the obtained glucose measurements to maintain euglycemia. A 4‐mL blood sample was collected at each of the following points for analysis of C‐peptide, human insulin, and IAsp levels: −30 and 0 minutes (before dosing) and 10, 20, 30, 40, 50, 60, 90, 120, 150, 180, 210, 240, 270, 300, 360, 420, and 480 minutes after dosing. The baselines for each of C‐peptide, human insulin, and IAsp were defined as the mean of the −30 and 0 minute (before dosing) measurements.

### Bioanalytical Methods

Whole blood glucose concentrations were tested with a glucose analyzer (Biosen C_line GP+, Neckar Healthcare, Co., Ltd., Magdeburg, Germany) using an automated glucose oxidase technique with a measuring range of 9 to 900 mg/dL and a high precision of 1.5%. Human insulin levels were determined using an ultrasensitive enzyme‐linked immunosorbent assay (ELISA) with monoclonal antibodies that have little or no cross‐reactivity to insulin analogs whose ranges of quantification were 0.0065 to 0.87 ng/mL for human insulin.^22^ C‐peptide levels were analyzed using a solid‐phase 2‐site ELISA method based on the direct sandwich technique in which 2 monoclonal antibodies are directed against separate antigenic determinants on the C‐peptide molecule with a measuring range of 0.06 to 9 ng/mL, with an intra‐assay coefficient of variation of 2.87% to 4.50%.^23^ IAsp concentrations were assessed by means of an ultra‐performance liquid chromatography–tandem mass spectrometry method^24^, ^25^ at Covance Laboratories in Shanghai that had no detectable cross‐reactivity with human insulin. Solid‐phase extraction was performed. Liquid chromatography separation was achieved using an Acquity UPLC CSH C18+ column (50 × 2.1 × 1.7 μm; Waters Corp., Milford, Massachusetts). A 20‐μL injection with a 0.60 mL/min flow of water with 0.1% of acetic acid (solvent A) and acetonitrile with 0.1% of acetic acid (solvent B) was used. The gradient elution was as follows: the initial 17.5% B was increased to 29.5% over 3.0 minutes, 29.5% to 50.0% over 0.5 minutes, 50% to 98% over 0.5 minutes, remained at 98% for 1.5 minutes, and returned to initial conditions over 0.5 minute. A mass spectrometer used in the positive ionization mode (ESI+) was used for the analysis of the compounds. The following conditions were found to be optimal for the analysis of aspart insulin: capillary voltage at 5.5 kV and source block temperature at 500^◦^C. The cone voltage and collision energy were adjusted to maximize the intensity of the protonated ion and to optimize the signal of aspart insulin: m/z 1133.4→971.8 and for the internal standard bovine insulin 1123.5→963.8. The lower limit of quantification was 0.2 ng/mL, the inter‐ and intravariability were 7.0% and 7.9%, respectively.

### Sample Sizes and Statistical Methods

The sample sizes of 22 for group A and 11 for group B yielded 91% power to detect a difference of 489.0 mg/kg in the area under the concentration‐time curve (AUC) of glucose infusion during the clamp (GIR AUC from time 0 to 8 hours [AUC_GIR,0‐8h_]) with known group standard deviations of 551.0 and 306.0, respectively, and a type I error of 5%. The time profiles of GIR and blood glucose were recorded during each clamp for individuals following the administration of IAsp. The GIR‐time profiles were used to calculate AUC_GIR,0‐8h_, maximum GIR (GIR_max_), and time of GIR_max_ (tGIR_max_) as the PD parameters. The PK parameters included maximum plasma concentration of IAsp (C_IAsp,max_), time of C_IAsp,max_, and the AUC of IAsp concentration from time 0 to 8 hours (AUC_IAsp,0‐8h_). The parameters mentioned above were calculated with PKSolver version 2.0.^26^ Homeostatic model assessment of insulin resistance was calculated as fasting glucose (mg/dL) × fasting insulin (mU/L) / 22.5.^27^ The endogenous insulin predicted by C‐peptide was determined on the basis of Owens's method^19^ using the following equation: [endogenous insulin] = *F* × C‐peptide, where *F* was the average of the ratio of insulin to C‐peptide at baseline. The clamp statistics included the coefficient of variation in blood glucose (CVBG), basal and clamped glucose, and the AUC of glucose excursion above and below the baseline.

The results were expressed as the mean ± standard deviation or median (25th, 75th percentile) for normally distributed and nonnormally distributed data, respectively. Normality was examined with Q‐Q plots. Unpaired Student's *t*‐test or the Mann‐Whitney *U* test was used to assess differences between groups A and B. Paired Student's *t*‐test was used to detect differences between the 2 methods for predicting endogenous insulin secretion, that is, the C‐peptide and the human insulin methods. Pearson's correlation test was used to determine the relationship between CVBG and the highest rate of increase of C‐peptide from baseline after injection of NovoRapid. Comparison of proportion of cases whose overall clamped glucose was below baseline was evaluated by Fisher's exact method. Since the 2 groups significantly differed in clamped glucose (*P* < .05, Table 1), an analysis of variance of AUC_GIR,0‐8h_ using clamped glucose as a covariate was conducted. A significance level of 5% (2‐sided) was used. All the data were analyzed by SPSS 22.0 (IBM, Armonk, New York) or Prism 8.4.2 (GraphPad Software, La Jolla, California).

**Table 1:** Subject Demographics and Statistics of the Euglycemic Clamp

Items	Group A	Group B	*P* Value
Number of subjects	22	11	…
Age, y^a^	23.7 ± 2.0	25.1 ± 2.0	.07
Height, cm^a^	173.1 ± 6.0	171.0 ± 4.4	.30
Weight, kg^a^	66.0 ± 8.4	61.6 ± 7.6	.15
BMI, kg/m^2^ ^a^	21.9 ± 1.8	21.0 ± 1.8	.16
Basal C‐peptide, ng/mL^b^	0.92 (0.83, 1.16)	0.90 (0.80, 1.25)	.74
Basal human insulin, ng/mL^b^	0.16 (0.13, 0.21)	0.18 (0.18, 0.28)	.25
Basal glucose, mg/dL^a^	80.6 ± 6.1	81.7 ± 4.7	.63
Clamped glucose, mg/dL^a^	80.3 ± 4.7	76.9 ± 4.5	<.01
Proportion of euglycemic clamps whose overall “clamped” BG was lower than baseline, %	22.73	90.91	<.01
CVBG, %^a^	4.31 ± 0.99	4.26 ± 1.01	.90
HOMA‐IR, mmol/L × mU/L^a^	0.80 ± 0.29	0.97 ± 0.34	.16
IAsp dosage, IU^a^	13.1 ± 1.75	12.4 ± 1.36	.21

BG, blood glucose; BMI, body mass index; CVBG, coefficient of variation of blood glucose; HOMA‐IR, homeostasis model assessment of insulin resistance; IAsp, insulin aspart.

^a^ Mean ± SD.

^b^ Median (25th, 75th percentile).

## Results

### Demographic and Disposition Data

Thirty‐three healthy male volunteers (22 in group A and 11 in group B) undergoing a manual euglycemic clamp study were enrolled in this study. The demographics of the subjects are presented in Table 1. No significant difference was detected in age, weight, height, body mass index, the dose of IAsp, or homeostatic model assessment of insulin resistance between groups A and B. There was no adverse reaction in any subject after injection of IAsp, and no adverse events were observed during the clamp procedures or the follow‐up period.

### Clamp Statistics in the 2 Groups

**Figure 1:**
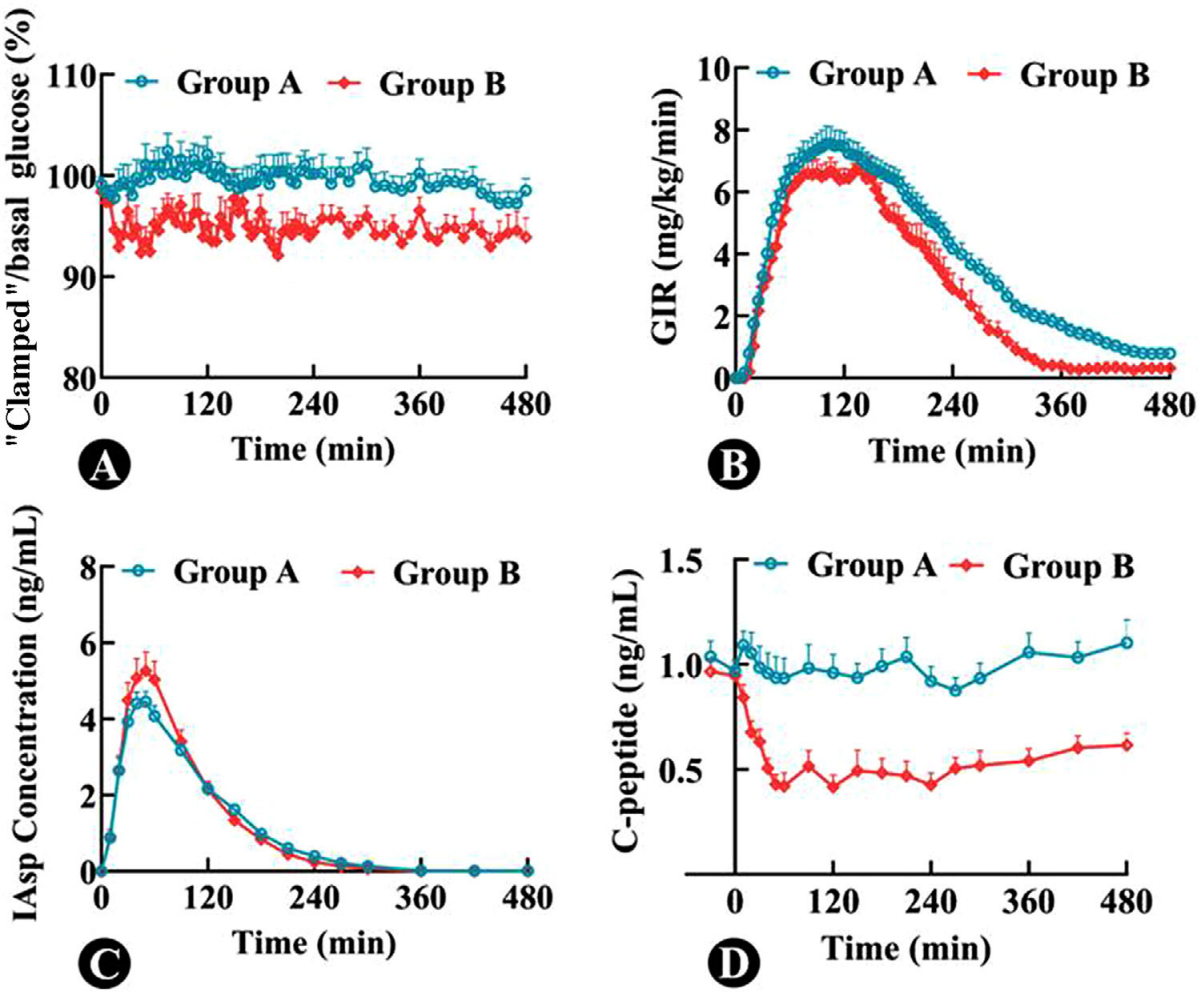
Time profiles of the ratio of “clamped” blood glucose to basal blood glucose (A), glucose infusion rate level (B), insulin aspart concentration (C), and C‐peptide level (D) during the euglycemic clamp (mean ± standard error).

As shown in Table 1, basal blood glucose and CVBG were comparable between groups A and B. The measured blood glucose during the clamp was higher in group A than in group B (*P* < .01). The overall clamped glucose concentrations in groups A and B were 99.7% and 94.9%, respectively, of baseline (*P* < .01; Figure 1A). The median AUCs of glucose excursion above baseline were 496 and 86.6% × minutes in groups A and B, respectively. The median AUCs of excursion under baseline were 843 and 2460% × minutes in groups A and B, respectively. As a result, nearly 37% (496/1339) of glucose excursions were above baseline in group A, whereas <4% (86.6/2546.6) of glucose excursions were above baseline in group B.

### Pharmacokinetic and PD Data of IAsp

Regarding the PD parameters (Figure 1B), there were no differences in GIR_max_ (8.46 ± 2.43 vs 7.90 ± 1.68 mg/kg/min; *P* = .50) and tGIR_max_ (111 ± 40.6 vs 113 ± 33.9 minutes; *P* = .92) between the 2 groups, whereas AUC_GIR,0‐8h_ was slightly higher in group A than that in group B (1815 ± 551 vs 1327 ± 306 mg/kg; *P*<.05). After correction for the overall clamped glucose, AUC_GIR,0‐8h_ remained higher in group A than in group B (1789 ± 107 vs 1380 ± 157 mg/kg; *P* = .048). GIR AUC from time 0.5 to 2 hours after correction for clamped glucose from 0.5 to 2 hours was 596 ± 38 and 520 ± 56 mg/kg in groups A and B, respectively. Regarding the PK parameters (Figure 1C), C_IAsp,max_ (4.72 ± 1.46 vs 5.53 ± 1.61 ng/mL; *P* = .16), time of C_IAsp,max_ (44.8 ± 11.0 vs 47.3 ± 11.0 minutes; *P* = .54), and AUC_IAsp,0‐8h_ (527 ± 97.1 vs 543 ± 97.2 ng/mL × minutes; *P* = .66) were comparable between groups A and B.

### C‐Peptide and Human Insulin Levels

**Figure 2:**
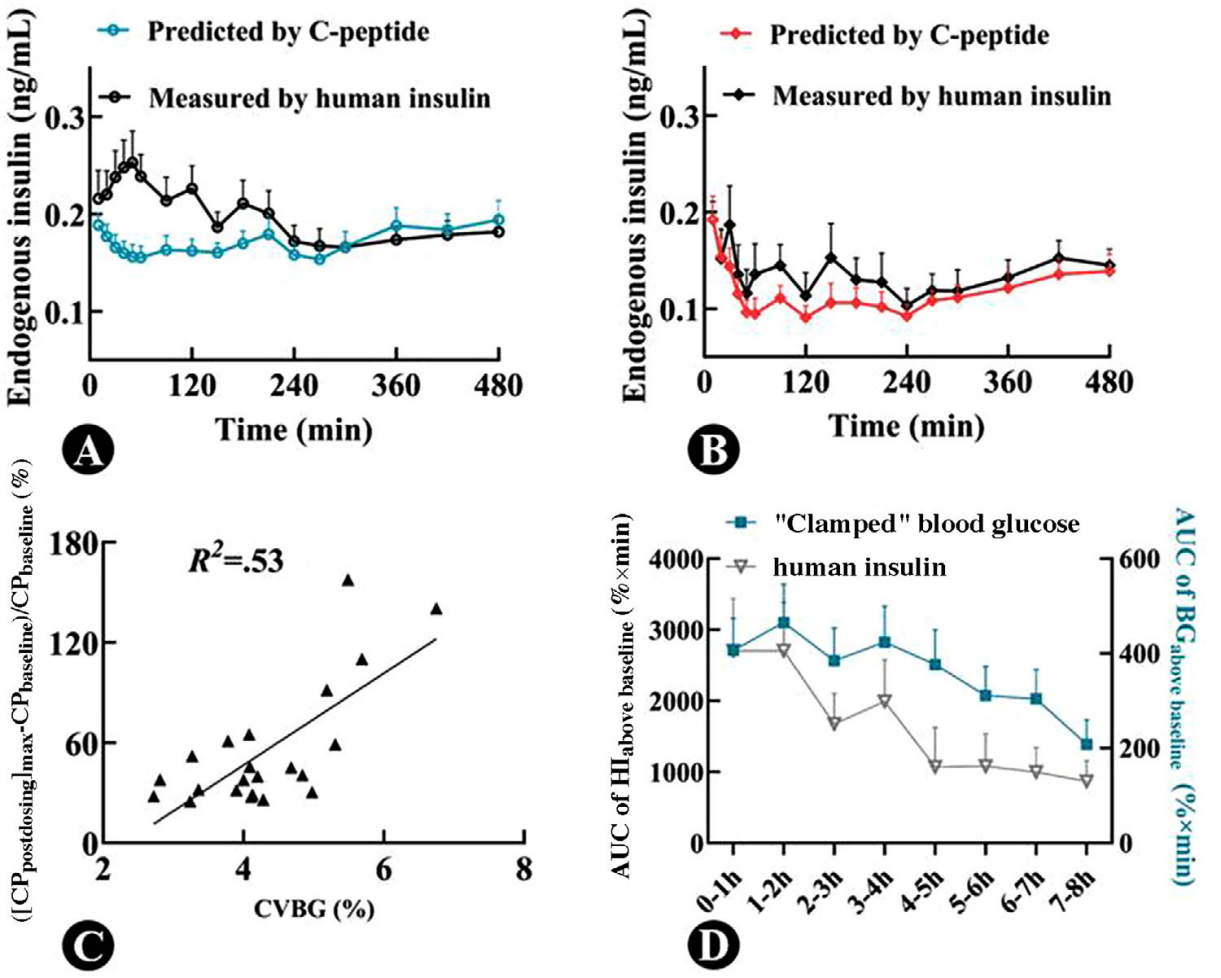
(A, B) Time profiles of endogenous insulin measured by human insulin or calculated by C‐peptide during the euglycemic clamp in groups A and B, respectively; (C) relationship between coefficient of variation of blood glucose (CVBG) and the highest rate of increase of C‐peptide after dosing (CP_postdosing_) from basal C‐peptide (CP_baseline_); (D) changes of the area under the concentration‐time curve (AUC) of increases of blood glucose and human insulin from their baselines per hour (mean ± standard error).

Neither C‐peptide nor human insulin level at baseline significantly differed between the 2 groups (Table 1). The C‐peptide levels after dosing are shown in Figure 1D. Endogenous insulin secretion predicted by C‐peptide and human insulin is shown in Figure 2A and B. The AUC of endogenous insulin predicted by C‐peptide from 0 to 8 hours was 80.6 ± 24.2 and 54.0 ± 21.1 ng/mL × minutes in groups A and B, respectively, and that predicted by human insulin was 91.0 ± 28.4 and 63.0 ± 27.3 ng/mL × minutes in groups A and B, respectively. The AUC_0‐8h_ of endogenous insulin predicted by C‐peptide was different from that predicted by human insulin (*P* = .033) in group A, whereas no significant difference between the methods was detected in group B (*P* = .14).

Of the 561 pairs of simultaneous serum C‐peptide and human insulin measurements collected after dosing, 316 had values that were both equal to or lower than their corresponding baselines, 138 had values that both higher than baseline, and the remainder had values that were inconsistent with each other (Table 2). Therefore, in 85.2% (138/162) of cases where C‐peptide level was elevated, human insulin was also increased, and in 79.2% (316/399) of cases where C‐peptide level was inhibited, human insulin was also suppressed.

**Table 2:** Frequencies of Different Patterns of Baseline‐Postdosing Relationships of Human Insulin and C‐Peptide Levels

Items	CP_postdosing_ > CP_baseline_	CP_postdosing_ ≤ CP_baseline_	Total
HI after dosing > HI baseline	138	83	221
HI after dosing ≤ HI baseline	24	316	340
Total	162	399	561

CP, C‐peptide; CPpostdosing, C‐peptide after dosing; CPbaseline, basal C‐peptide; HI, human insulin.

### Effects of Blood Glucose Fluctuation on the Levels of C‐Peptide and Human Insulin

The relationship between CVBG and the probability of C‐peptide levels being above baseline during the euglycemic clamp is shown in Figure 2C. All CP_postdosing_ values were below baseline when CVBG was <2%. Interestingly, as CVBG increased, the probability of CP_postdosing_ being higher than baseline increased, and there was a positive correlation between the 2 measures (*R* ^2^ = .53). The relationship between the AUC of clamped blood glucose higher than the basal blood glucose per hour and the AUC of human insulin higher than baseline per hour is shown in Figure 2D. As the AUC of blood glucose above baseline decreased, the AUC of human insulin higher than baseline also decreased, with the 2 parameters exhibiting the same trend.

## Discussion

The euglycemic glucose clamp technique has been regarded as the best available method for the assessment of PK/PD values of study insulin and its analogs.^28^ Sufficient suppression of endogenous insulin secretion in such assessment is of considerable importance, as it affects the precision of PK/PD assessments of new insulin preparations in the healthy. C‐peptide, cleaved from the proinsulin molecule in islet cells, is released into the circulation in amounts equimolar to insulin,^29^ and the hepatic extraction of C‐peptide is negligible. Therefore, C‐peptide is usually measured in parallel to insulin concentrations during euglycemic clamp studies to evaluate whether endogenous insulin secretion is inhibited. The half‐life of C‐peptide^30^ is longer than that of human insulin such that it cannot correct for rapid changes due to poor clamp technique.^31^ In the present study, human insulin was measured by a reliable ELISA method with little cross‐reactivity with IAsp. Therefore, the endogenous insulin represented by human insulin might be more similar to the true situation. We considered human insulin to be roughly equivalent to endogenous insulin secretion. The study revealed that the method using C‐peptide to predict endogenous insulin secretion had a sensitivity of 85.2% and a specificity of 79.2%. Increased C‐peptide levels can thus be regarded as a marker of insufficient inhibition of endogenous insulin.

In general, clamp studies aimed at evaluating short‐acting insulin preparations require that the CVBG not exceed 10%, whereas those aimed at evaluating long‐acting insulin preparations require that it not exceed 5%.^32^ The results of the present study showed that as the CVBG increased, the distance of C‐peptide above baseline increased (Figure 2C), and there was a positive correlation between the 2 measures. These findings indicate that large fluctuations in blood glucose may be one factor responsible for insufficient suppression of endogenous insulin secretion. However, since the overall CVBG values of group A and group B were comparable in this study, we speculate that there may be other factors associated with uninhibited endogenous insulin secretion. The blood glucose is recommended to be clamped below the subject's fasting glucose in healthy volunteers^33^ and be controlled to within ±10% of the target value. Other researchers have reported that blood glucose could be clamped at the individual's basal level.^6^, ^7^, ^8^, ^9^, ^10^, ^11^, ^12^, ^13^ Based on the AUCs of glucose excursion in our study, nearly 37% of excursions were above baseline when the clamped glucose was maintained around baseline (group A), whereas <4% of glucose excursions were above baseline when the clamped glucose was ≈5% below baseline (group B). Clamped glucose around baseline increased the possibility of over‐baseline glucose excursion, which might stimulate endogenous insulin secretion.^34^, ^35^

Insufficient information is available to evaluate the accuracy of insulin PK/PD data obtained in the clamp in the context of an elevated postdosing C‐peptide level. Some researchers have suggested if the C‐peptide level is increased by >200 pmol/L from baseline after dosing, the data are not suitable for analysis.^36^ One possible reason for the difference in AUC_GIR,0‐8h_ after correction for clamped glucose between the 2 groups in this study is the difference in C‐peptide level between the 2 groups. Another possible explanation is the unequal extent of suppression of hepatic glucose production (HGP) between the groups due to the absence of a continuously high blood insulin level. Much stronger, almost complete, suppression of HGP has been observed at serum insulin levels >40 uU/mL.^32^ We calculated the AUC of GIR for the period from 0.5 to 2 hours, when insulin level was high, to minimize the interference due to HGP. The GIR AUC from time 0.5 to 2 hours after clamped glucose correction differed by ≈14.6% between the 2 groups. This finding indicates that if a subject's endogenous insulin secretion is not sufficiently inhibited, it will contribute to the observed GIR. Furthermore, we found that elevated postdosing C‐peptide level was a marker of inappropriately excessive glucose infusion.

In studies in which specific assays for the tested insulin are lacking, C‐peptide was always used to correct endogenous insulin.^37^, ^38^ Some researchers^39^ still doubted the use of C‐peptide to correct endogenous insulin because some PK parameters were not consistent (eg, a peak of glargine in plasma occurring at 12 hours^40^ differs from those observed in subjects with type 1 diabetes where the increase occurs between ≈3 and 6 hours after the first injection^41^ and at steady state^42^). Owens^19^ reported the exogenous insulin estimated from C‐peptide was nearly the same as what was true when C‐peptide was well inhibited. Moreover, the results of the present study showed that in the absence of endogenous insulin inhibition (group A), the value of total endogenous insulin predicted using C‐peptide was significantly different from that predicted by human insulin, whereas no such significant difference was detected when postdosing C‐peptide was inhibited (group B). Therefore, estimates of the total exogenous insulin exposure might be inaccurate without enough suppression of C‐peptide.

In conclusion, unsuppressed endogenous insulin may contribute to observed GIR, and insulin PK values estimated from the C‐peptide correction method may not be accurate in the absence of endogenous insulin suppression. The interpretation of the PD and PK data corrected by C‐peptide should be done with caution when endogenous insulin is not inhibited, and perhaps a sensitivity analysis of data excluding partial clamps with insufficient C‐peptide inhibition is needed.

## Funding

The data were collected from 2 clinical trials (CTR20160095 and CTR20180517; more information is available on the website http://www.chinadrugtrials.org.cn/) funded by Zhuhai United Laboratories Co., Ltd, and Yichang HEC Changjiang Pharmaceutical Co., Ltd, respectively, but neither were involved in the execution of the study or the writing of the article.
